# Annual Meeting of the Academy of Dental Science

**Published:** 1868-11

**Authors:** 


### ARTICLE IV.

### *Annual Meeting of the Academy of Dental Science.*

The annual meeting of the American Academy of Dental Science was held September 28th, at the rooms of the Suffolk District Medicinal Society in Temple place, Boston, the President, Dr. E. T. Wilson, in the chair, and Dr. E. N. Harris officiating as Secretary. The meeting was quite fully attended, and after the transaction of business of an unimportant character, the following board of officers for the ensuing year was elected: President—Daniel Harwood, M. D. Vice President—E. T. Wilson, M. D. Secretary (corresponding and recording)—E. N. Harris, D. D. S. Librarian —John Clough, M. D. Censors—E. G. Tucker, M. D., D. M. Parker., M. D., and J. L. Williams, M. D.

Interesting and pertinent remarks connected with dental science were then made by Dr. Daniel Harwood, of Boston, Dr. J. H. Foster, of New York, and others. The annual address was then delivered by Dr. E. T. Wilson. After welcoming his fellows to the hospitalities of the occasion, and congratulating them upon the pleasures proffered by an anniversary, he said that it was good for the devotees of a common pursuit, the professors of an important and useful science, the cultivators of an ingenious and beautiful art, at stated periods to gather together, that each may compare his own experience with that of his fellow, and communicate, in a spirit of professional courtesy, whatever of useful novelty may have fallen under his observation, and in return receive the fresh results of recent labor in different parts of the country. We meet, he said, for the interchange of personal courtesies, for the expression of personal good wishes, and for the renewal of old friendships. We also meet to tender to the venerable leaders of the profession our congratulations upon its steady and brilliant advancement. We meet also to snatch an hour from the work of the laboratory and the weariness of the office, and by a short relaxation of the mind and recreation of the body to gather strength for the duties and trials which are before us.

After alluding at some length to the origin and growth of the profession, stating in this connection that dentistry was the offspring of the refined civilization of the last century, he passed on to the consideration of the subject of membership, of the "American Academy of Dental Science." Alluding to the Academy he then said that it had flourished as well as it could, and that it seemed to be getting along tolerable well, and considering what dentistry was half a century ago, and what it is now, and considering also how little fraternal encourgement they had received from members of the medical profession, all that he could do was to point to the work of their hands and ask for an honest verdict. Dentistry has been created as a science almost in our day, and it has been created by dentists. No liberal pursuit has made more rapid advancement, and this too under most discourageing circumstances. Patient observation and intelligent experiment has done the work. He accordingly claimed that the profession already has status, respectability, cultivation knowledge, and a literature of its own, and no more than its fair proportion of quacks. There are of course dexterous and clumsy operators, but some kind of work, we all admit it, is necessary to do—and when we do make a botch we are not permitted to hide away our failure in a cemetery, for it keeps walking about grinning horribly a ghastly smile, and advertising our incompetency.

But this academy has its own standard of professional acquirement, which it means to maintain, notwithstanding the fact that a new process of grinding out dentists has at last arrived. Now, it seems, that young men are to be lectured into dexterity, taught the art of extracting in "twelve easy lessons," and the whole use of the instruments by a faculty of erudite professors. This he regarded as all wrong, and part of the humbug of the day. What the dentist needs is an educated hand, eye, and heart. Good work takes time, and requires practice. He then went on to speak of the Academy as an auxilliary in promoting the usefulness and sustaining the respectability of the profession. He condemned the practice of resorting to quackish expedients for the purpose of making money, saying that the largest fortune was not worth attaining in that way—manliness is of more value than money, and self-respect should not be parted with for silver, or a good reputation for gold. A popularity rapidly acquired is apt to be speedily lost. Patient effort only was the lasting popularity. He was proud to say that there were reputations in the profession that had not been cheaply won, and cannot be easily lost. Such men have the confidence of the public and the respect of every honest dentist, and long may they remain to dignify the profession. Claiming as we do that ours is a progressive profession, we may reasonably look forward to new discoveries, and to improvements such as are now hardly dreamed of. A good dental operator, said the speaker, should be a "permanence," and have his fixed and abiding "patients"—not "patrons," for nobody patronizes, because for fair work he pays an adequate fee—pays for time, toil, thought, skill and experience. To deceive the trusting, to delude the confiding, and make suffering the sport of avarice, is meanness, cruelty and falsehood combined. Yet this is what many who proclaim themselves dentists are doing every day. Here followed a chapter of remarks on the quacks and quackery of the profession that was quite severe, and which (the quackery) he trusted would soon be put down forever. Yet it can be done only by waging an unending war against it. In closing his remarks, he alluded to the subject of professional character and dignity, and the necessity of maintaining a high standard in this respect. Every member should be sensitive on the subject of "professional honor and character." Each of us, said the speaker, has a fraternal pride in the success of every other. By the recognition of this truth we shall prove ourselves the servants of knowledge and the fortunate benefactors of the race.

At the conclusion of the address, which was most happily received, the company adjourned to the Revere House, and partook of their annual dinner, Dr. E. T. Wilson presiding. Prayer was offered by Rev. Geo. D. Johnson, rector of St. Marks Church. The repast being over, after-dinner remarks were made by his Honor Mayor Shurtleff, Dr. Daniel Harwood, Dr. J. H. Foster, of N. Y., Rev. Mr. Johnson, Drs. J. L. Williams, E. G. Tucker, B. S. Codman, C. H. Frothingham, Esq., of Boston, and H. F. Bishop, of Worcester. Among the invited guests were Dr. Winslow Lewis, of Boston, and Dr. Gage of the Dental Fraternity of Paris, and others. At the business meeting Dr. Daniel Harwood was appointed to deliver the next annual address.

